# Characterization of the oral microbiome and gut microbiome of dental caries and extrinsic black stain in preschool children

**DOI:** 10.3389/fmicb.2023.1081629

**Published:** 2023-03-31

**Authors:** Luoyuan Zheng, Tingting Cao, Puling Xiong, Yulian Ma, Limin Wei, Jianfeng Wang

**Affiliations:** ^1^School and Hospital of Stomatology, Wenzhou Medical University,, Wenzhou, China; ^2^Department of Preventive Dentistry, School and Hospital of Stomatology, Wenzhou Medical University, Wenzhou, China; ^3^Department of Orthodontics, School and Hospital of Stomatology, Wenzhou Medical University, Wenzhou, China

**Keywords:** extrinsic black stain, dental caries, oral microbiome, gut microbiome, primary teeth, risk factor

## Abstract

**Introduction:**

A lower prevalence of dental caries (hereafter termed “caries”) has been observed in children with dental extrinsic black stain (EBS).

**Methods:**

We investigated the epidemiologic characterization of EBS and explored the possible role of the oral microbiome (OM) and gut microbiome (GM) in EBS formation and caries prevention. In an epidemiologic survey, 2,675 children aged 3–6 years were included. Thirty-eight of these children (7 children had both caries and EBS, 10 had EBS only, 11 had caries only, and 10 were healthy children) were recruited for 16S rRNA sequencing and collection of samples of supragingival plaque and feces. Collected plaque samples were divided into four groups: BCP (EBS+, caries+), BP (EBS+, caries−), CP (EBS−, caries+), and P (EBS−, caries−). Fecal samples were also divided into four groups: BCF (EBS+, caries+), BF (EBS+, caries−), *CF* (EBS−, caries+), and F (EBS−, caries−).

**Results:**

EBS was observed in 12.10% of this population. Children with EBS had a significantly reduced prevalence of caries and a lower mean value of decayed–missing–filled teeth (dmft; *p* < 0.01). According to analyses of dental plaque, the P group had the most complex microbiome. The BCP group exhibited greater operational taxonomic unit (OTU) richness but a reduced evenness compared with the BP group, and the CP group showed greater OTU richness than the BP group. At the genus level, higher abundance of *Actinomyces* and *Cardiobacterium* species was observed in the BCP group. Higher abundance of *Lautropia* and *Pesudopropionibacteriumin* species was observed in the BP group compared with P and CP groups, respectively (*p* < 0.05). *Veillonella* species were significantly more common in P and CP groups than in BP groups, whereas *Porphyromonas* and *Fusobacterium* species were more common in the CP group (*p* < 0.05). With regard to the GM, the *CF* group exhibited greater OTU diversity than the BF group. The GM in the BCF group exhibited the most complex relationships across all fecal groups. GM groups could be distinguished by various unique biomarkers, such as *Escherichia* and *Shigella* species in the BCF group, *Agathobacter* and *Ruminococcus* species in the *CF* group, *Lactobacillus* species in the BF group, and *Roseburia* species in the F group. Our results suggest that EBS is a possible protective factor against early-childhood caries. Dental plaque and the GM may be relevant to EBS in primary dentition.

## 1. Introduction

According to the World Health Organization, dental caries (hereafter termed “caries”) is the third-leading major disease that seriously impacts human health (after cancer and cardiovascular disease; Lei et al., 2021). Consistently, a significant amount of research has been conducted on its development and etiology.

As a common symptom in primary teeth, extrinsic black stain (EBS) has attracted significant attention. EBS is defined as a dark-pigmented extrinsic substance on the buccal and/or lingual surfaces of primary and permanent teeth near the gingival margin (Supplementary Figure 1A; Watts and Addy, 2001). EBS has been shown to be a protective factor against caries in early childhood. However, the detailed mechanisms at play in this relationship are not known.

Zhang et al. (2017) showed that the iron content in black stains was greater than that in the plaque of the control group. However, their results may be attributable to use of an iron scoop. Parnas et al. (2013), using graphite, found no difference between EBS and other types of plaque. Aysun et al. (2012) documented a significantly higher capacity for salivary buffering and the calcium concentration and a lower flow rate in children with black tooth stains compared with those without black tooth stains.

Some studies have reported that special features of the microbiota differ between EBS groups and control groups; for example, the black-stain microbiome has less species diversity than that of white-plaque microbiome. The five most abundant genera found in EBS are *Capnocytophaga*, *Leptotrichia*, *Fusobacterium*, *Corynebacterium*, and *Streptococcus* (Colak et al., 2013). In another study, samples from the EBS group contained greater numbers of *Actinomyces naeslundii* and lower numbers of *Fusobacterium nucleatum* and *Lactobacillus* species compared with samples from the control group (Heinrich-Weltzien et al., 2014). One review determined the EBS microflora to be dominated by *Actinomyces* species (Zyla et al., 2015).

Several hundred trillion microbiome cells live in harmony in the human body, including in the skin, oral cavity, and gastrointestinal tract. The gut microbiome (GM; also known as “gut microbiota,” or “gut flora”) includes >400 resident floras. There are 10-times more GM cells than all other human cells collectively. The oral microbiome (OM; also known as “oral microbiota”) ranks second in diversity and abundance to that of the GM (Zhao et al., 2020). Research suggesting that the GM has a facilitating role in the relationship between the stress response, inflammation, depression, and anxiety is emerging (Peirce and Alvina, 2019). Recent studies have shown that bacteria inhabiting the oral cavity can translocate to the gut and drive GM dysbiosis (Heidrich et al., 2021). Knowledge of an association between the OM and various gastrointestinal diseases has existed since OM research first began. Kitamoto et al. (2020) found that irritable bowel syndrome, inflammatory bowel disease, and colorectal cancer were strongly linked to the OM. Low diversity and richness in the GM are clearly associated with periodontitis. Regardless of periodontal status, we found that a large number of intestinal microorganisms correlate with periodontal inflammation and destruction (Baeta et al., 2018). Shishniashvili and colleagues determined that the GM facilitates demineralization of dental hard tissue, thereby enabling the formation of caries (Shishniashvili et al., 2018).

Dental plaque and saliva are easier to collect than samples of the intestinal microbiome. Hence, many studies have focused on the relationship between the OM and EBS, and few studies have assessed the intestinal microbiome. Nevertheless, some studies on the connections between the intestinal microbiome and other systemic diseases have been done.

Here, we focused on GM characterization in children with and without EBS. Specifically, we sought to provide evidence for therapeutic approaches and preventive strategies by assessing the epidemiologic features of EBS and using 16S rRNA sequencing to analyze GM variations associated with EBS, caries, and health in children aged 3–6 years with primary dentition.

## 2. Materials and methods

### 2.1. Study participants

The study protocol was approved (WYKQ2022008) by the Ethics Committee of the School and Hospital of Stomatology affiliated to Wenzhou Medical University (Wenzhou, China). The study was conducted on 28 June 2022. Written informed consent was obtained from the parents of all children.

Using random cluster sampling, we selected 15 preschools in Wenzhou (a city located in Zhejiang Province in China). We calculated the sample size using the following formula:

*n* ≥ *t*^2^*p* (1 − *p*)/*d*^2^.

where *t* is the confidence level at 95%, *p* is the estimated prevalence of EBS (based on a previous pilot survey, prevalence was 12.1%), and d is the margin of error.

The calculated sample size was ≥191. In kindergartens, a dentist with specialized training and specialized equipment conducted oral examinations according to guidelines established by the World Health Organization (Colak et al., 2013; World Health Organization, 2013). The epidemiology of caries was estimated based on the DMFT Index (which assesses decayed, missing, and filled teeth). EBS was evaluated according to the existence of dark lines parallel to the gingival margin or an incomplete merger of dark dots, which rarely extend past the cervical one-third of the crown surface.

Based on the results of the clinical examination, four groups of 40 participants (10 participants/group) were chosen from among 2,675 children. Some samples were excluded because some children had taken antibiotics (or any other medication) during our study. Thirty-eight participants were included but just 74 samples were collected finally. Participants had not taken antibiotics (or any other medication) within 3 months before this study. Also, they had not undergone tooth polishing ≤3 months before the study and had no intrinsic black stains.

Collected plaque samples were divided into four groups: BCP (EBS+, caries+), BP (EBS+, caries−), CP (EBS−, caries+), and P (EBS−, caries−). Fecal samples were also divided into four groups: BCF (EBS+, caries+), BF (EBS+, caries−), *CF* (EBS−, caries+), and F (EBS−, caries−). The EBS tooth and caires tooth number were required >5 in corresponding group. The inclusion and exclusion criteria for our study are listed in Tables 1, 2, respectively.

**Table 1:** Inclusion criteria.

Group 1	Group 2	Group 3	Group 4
(*n* = 7)	(*n* = 10)	(*n* = 11)	(*n* = 10)
>5 teeth with EBS	>5 teeth with EBS	Age = 3–6 years	Age = 3–6 years
Age = 3–6 years	Age = 3–6 years	Dmf > 5	Free of systemic diseases
Free of systemic diseases	Free of systemic diseases	Free of systemic diseases	Free of EBS, caries, gingivitis, or periodontitis
Dmf > 5	Free of caries, gingivitis, or periodontitis	Free of EBS	

All members of each group had primary dentition and written informed consent was obtained from parents.

**Table 2:** Exclusion criteria.

Received antibiotics ≤3 months before study commencement
Underwent tooth polishing ≤3 months before study commencement
Had any intrinsic black stain

### 2.2. Sample collection

Children were asked not to brush their teeth in the morning and not to consume any food or drink for ≥2 h but to clean any debris in their mouth by gargling with clean water before sample collection. Samples of supragingival plaque and EBS were collected from the buccal surfaces of upper molars and lower anterior teeth using sterile swabs and curettes at 09:00–10:00 am. When collecting fecal samples, sterile containers were used to scoop-out a middle portion of the stool isolated from air (Supplementary Figures 1B,C). One portion of plaque was placed in axenic medium; then, this medium was placed in an incubator at 37°C for 48 h. We analyzed the results subsequently (Supplementary Figure 1D; Table 3). The remaining plaque and all stool samples were stored at −80°C for further studies. In all cases, sample collection took <4 h.

**Table 3:** Caries activity test (CAT) scores.

CAT score (points)	Color	pH
0	Purple	>6
1	Blackish-green	5.9–5.6
2	Grass-green	5.5–5.1
3	Light-green	5.0–4.9
4	Yellow-green	4.8–4.6
5	Yellow	<4.5

### 2.3. Illumina MiSeq sequencing

Purified amplicons were pooled in equimolar and paired end-sequenced on the MiSeq™ PE300 platform (Illumina, San Diego, CA, USA) according to standard protocols set forth by Majorbio Bio-Pharm Technology (Shanghai, China).

### 2.4. DNA extraction and polymerase chain reaction amplification

DNA (deoxyribonucleic acid) from the OM and GM was extracted from plaque and fecal samples, respectively, using the E.Z.N.A.® Soil DNA kit (Omega Biotek, Norcross, GA, USA) according to manufacturer instructions. DNA extracts were checked on 1% agarose gel. The concentration and purity of DNA were determined using an UV–Vis spectrophotometer (NanoDrop™ 2000; Thermo Fisher Scientific, Waltham, MA, USA). The hypervariable region V3–V4 of the bacterial 16S rRNA gene was amplified with the primer pairs 338F (5′-ACTCCTACGG GAGGCAGCAG-3′) and 806R (5′GGACTACHVGGGT WTCTAAT-3′) by a PCR thermocycler (GeneAmp™ 9,700; Applied Biosystems, Foster City, CA, USA). PCR amplification of the 16S rRNA gene was undertaken using the following cycle: initial denaturation at 95°C for 3 min, followed by 27 cycles of denaturing at 95°C for 30 s, annealing at 55°C for 30 s, and extension at 72°C for 45 s; then, a single extension at 72°C for 10 min, ending at 4°C. Each PCR mixture contained 5× TransStart® FastPfu buffer (4 μL; TransGen Biotech, Beijing, China), 2.5 mM of dNTPs (2 μL), 5 μM of forward primer (0.8 μL), 5 μM of reverse primer (0.8 μL), TransStart® FastPfu DNA polymerase (0.4 μL) (TransGen Biotech), template DNA (10 ng), and double-distilled H_2_O (≤20 μL). PCRs were carried out in triplicate. The PCR product was extracted from 2% agarose gel, purified using a DNA gel extraction kit (Axygen Biosciences, Union City, CA, USA) according to manufacturer instructions, and quantified using the Quantus™ fluorometer (Promega, Madison, WI, USA).

### 2.5. Processing of sequencing data

Raw 16S rRNA gene-sequencing reads were demultiplexed, quality-filtered by fastp 0.20.0 (Chen et al., 2018), and merged by FLASH 1.2.7 (Magoč and Salzberg, 2011) according to three main criteria. First, 300-bp reads were truncated at any site receiving an average quality score < 20 points over a 50-bp sliding window, and truncated reads shorter than 50 bp and any reads containing ambiguous characters were discarded. Second, only overlapping sequences longer than 10 bp were assembled according to the overlap. (The maximum mismatch ratio of the overlap region was 0.2) Reads that could not be assembled were discarded. Finally, samples were distinguished according to their barcode and primers, and the sequence direction was adjusted *via* exact barcode matching, with a 2-nucleotide mismatch allowed in primer matching.

Operational taxonomic units (OTUs) with a 97% similarity cutoff (Stackebrandt and Goebel, 1994; Edgar, 2013) were clustered using UPARSE 7.1 (Edgar, 2013), and chimeric sequences were identified and removed. The taxonomy of each OTU-representative sequence was analyzed by RDP Classifier 2.2 (Wang et al., 2007) against the 16S rRNA database (e.g., Silva version 138) using a confidence threshold of 0.7.

### 2.6. Bioinformatics analysis

Characteristic sequences were split at various classification levels (from phylum to OTU). Bacterial diversity in terms of richness was assessed with α and β indices. The species abundance of each sample was counted at different taxonomic levels. The community composition was studied intuitively by a series of visualization methods, including histograms and heatmaps. Based on the data obtained on community abundance, rigorous statistical methods were used to test the hypothesis of species between different groups (or samples) of microbial communities, evaluate the significance level of differences in species abundance, and obtain the species with significant differences between groups (or samples). The association of microbial-community structure with affecting factors was evaluated by variation partitioning along with distance-based redundancy analysis (db-RDA). Co-occurrence network analysis can be used to show the distribution between samples and species. Through the analysis of information on species abundance between different samples, the co-existence relationship between species in environmental samples can be obtained, and similarities and differences between samples can be highlighted.

### 2.7. Questionnaire

The parents of participants completed standard questionnaires which asked questions about whether their children had anemia, their dietary habits (e.g., frequency and dose of drinking milk powder, brand of milk powder), and oral hygiene (frequency of visiting a physician and tooth-brushing). The information requested also included sociodemographic characteristics such as age, as well as the education, income, and the awareness of oral healthcare of their parents.

### 2.8. Statistical analyses

The chi-squared test was used to compare differences in the prevalence of EBS and caries between different groups. The Mann–Whitney *U*-test (two groups) and Kruskal–Wallis test (≥3 groups) were employed to compare dmft values among different groups. Spearman’s rank correlation test was also used to assess differences in the caries activity test (CAT) scores between groups using Cariostat® (Sankin, Tokyo, Japan).

## 3. Results

### 3.1. Epidemic-related information on EBS and caries

A total of 2,675 children (3–6-years) underwent oral examinations during this study. Children with mixed dentition, who were absent from school, or who had incomplete examinations were excluded. Finally, data from 2,331 children were included in our final analysis.

The prevalence of caries in the whole study population was 63.88% (1,489/2,331). A higher caries prevalence was found among molar teeth and maxillary anterior teeth compared with that in any other tooth type. EBS was observed in 12.10% (282/2,331) of this population (Table 4). The teeth and surfaces most afflicted by EBS were the mandibular anterior teeth and their lingual surfaces, respectively (Supplementary Figure 1E). Compared with those in the control group, children with EBS had a significantly lower prevalence of caries (*p* < 0.01). The mean dmft value was 2.46 ± 3.49 in the EBS group and 3.83 ± 4.35 in the control group, respectively, and this difference was significant (*p* < 0.01). The prevalence of EBS showed an increasing linear trend with age (*p* < 0.01). Inverse correlations between the prevalence of EBS and caries, and between EBS and the mean dmft value, were found (*p* < 0.01; Table 5).

**Table 4:** Caries and extrinsic black stain (EBS) patterns according to age group.

Age (years)	Sex	Number	EBS prevalence	Caries prevalence	Mean dmft score^b^
3–4	M	226	8.41%	51.77%	2.23 ± 3.40
	F	206	11.65%	45.15%	2.09 ± 3.40
Total		432	9.95%	48.61%	2.16 ± 3.41
4–5	M	578	13.15%	62.28%	3.43 ± 4.26
	F	450	9.33%	65.33%	3.78 ± 4.30
Total		1,028	11.48%	63.62%	3.58 ± 4.28
5–6	M	440	13.64%	72.05%	4.56 ± 4.48
	F	328	13.41%	70.12%	4.15 ± 4.26
Total		768	13.54%	71.22%	4.38 ± 4.39
6–7	M	65	20.00%	72.31%	5.08 ± 5.11
	F	38	10.53%	81.58%	4.89 ± 4.40
Total		103	16.50%	75.73%	5.01 ± 4.86
Total		2,331	12.10%	63.88%	3.65 ± 4.28
*r*_s_			0.048	0.164	0.202
*p* ^a^			0.021^*^	<0.01^**^	<0.01^**^

a Comparison among different age groups, ^*^p < 0.05, ^**^p < 0.01, ^***^p < 0.001.

b Mean ± SD.

**Table 5:** Association between the mean dmft value\caries prevalence and extrinsic black stain (EBS) in 2,331 children in Wenzhou, China.

	Groups		
Item	EBS	Without EBS	*p*^a^
	*n* (%)	*n* (%)	
Caries prevalence (%)	282 (51.77%)	2049 (65.50%)	<0.01^**^
Mean dmft ± SD	2.45 ± 3.50	3.81 ± 4.35	<0.01^**^

^a^Comparison between preschool children with and without EBS, ^**^p < 0.01.

Extrinsic black stain was more prevalent in children whose parents had a bachelor’s degrees or above, knowledge of Fluor Protector (Ivoclar, Schaan, Liechtenstein) for teeth (*p* < 0.05), and more regular oral checkups (*p* < 0.01; Supplementary Table 1). Children who consumed Cow & Gate™ milk powder (Danone, Hoofddorp, Netherlands) had a higher prevalence of EBS compared with children who drank any other type of milk powder (*p =* 0.029). The prevalence of EBS in children who received oral treatment often (*p* < 0.01) was also greater (Supplementary Table 2).

### 3.2. Cat scores

Caries activity test scores of 0 and 1 point(s) were regarded as suggestive of a low risk of caries. In contrast, CAT scores of 2 and 3 points indicated a medium risk of caries, while those of 4 and 5 points indicated a high risk of caries. A positive correlation between the CAT score and mean dmft value was found (*p =* 0.023; Table 6).

**Table 6:** Relationship between the caries activity test (CAT) score, caries prevalence, and mean dmft value.

CAT score	*n*	Caries prevalence (%)	Mean dmft ± SD
Low	18	0.39	2.28 ± 4.17
Middle	37	0.43	2.62 ± 3.55
High	13	0.77	6.30 ± 4.92
*r*_s_		0.233	0.275
*p*		0.056	0.023^*^

* CAT score was positively correlates with the mean dmft value, ^*^p < 0.05.

### 3.3. Sequencing

#### 3.3.1. Sequencing characteristics

Seventy-four samples were collected for further analyses and divided into eight subgroups: four for dental plaque and four for feces. The subgroups for dental-plaque samples were BCP (EBS+, caries+, *n* = 7), BP (EBS+, caries−, *n* = 10), CP (EBS−, caries+, *n* = 11), and P (EBS−, caries−, *n* = 10). The subgroups for feces samples were BCF (EBS+, caries+, *n* = 7), BF (EBS+, caries−, *n* = 10), *CF* (EBS−, caries+, *n* = 10), and F (EBS−, caries−, *n* = 9).

**Figure 1:**
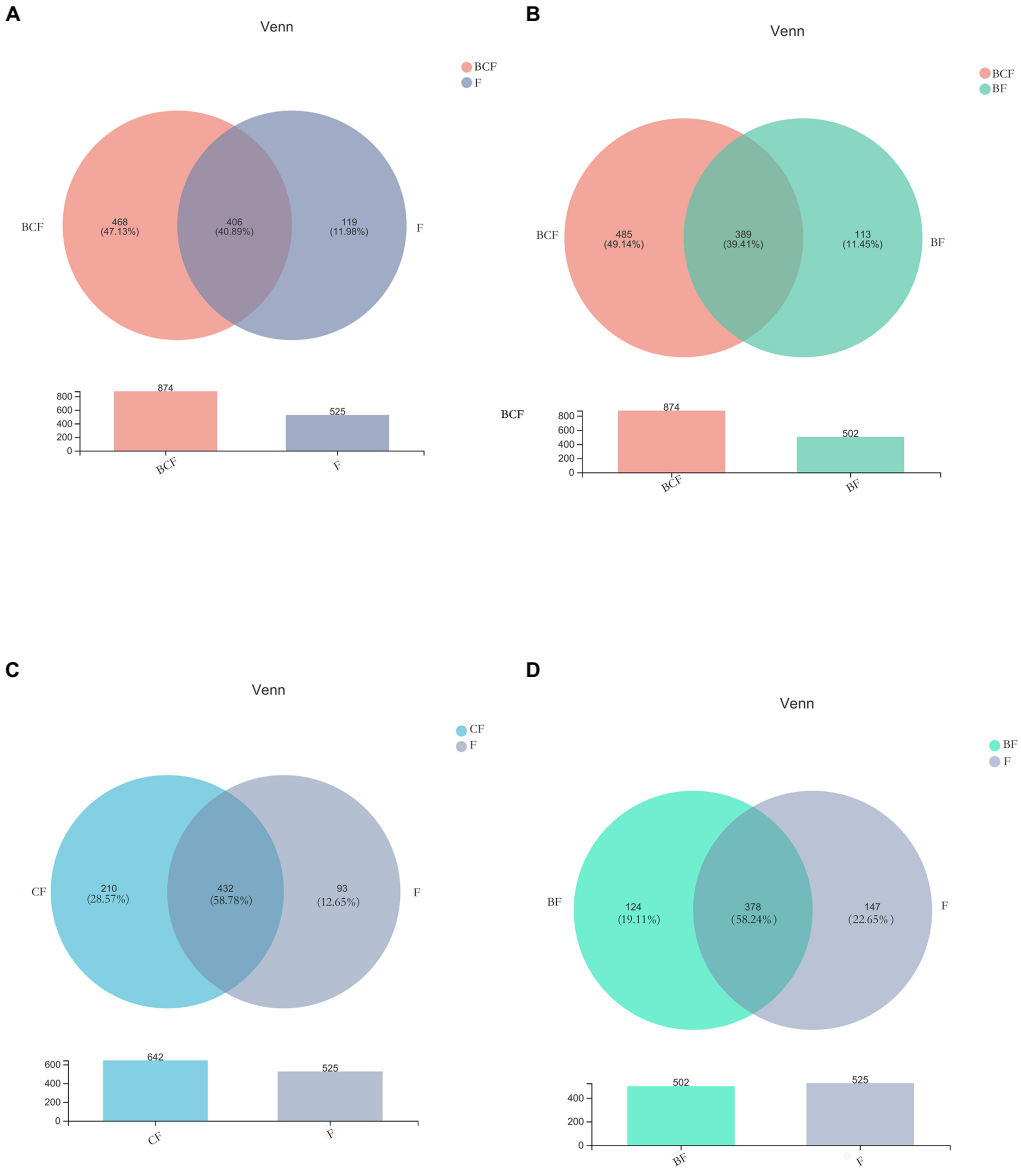
Venn diagrams depicting operational taxonomic unit (OTU) distributions in **(A)** BCF and F, **(B)** BCF and BF, **(C)** *CF* and F, and **(D)** BF and F groups.

**Figure 2:**
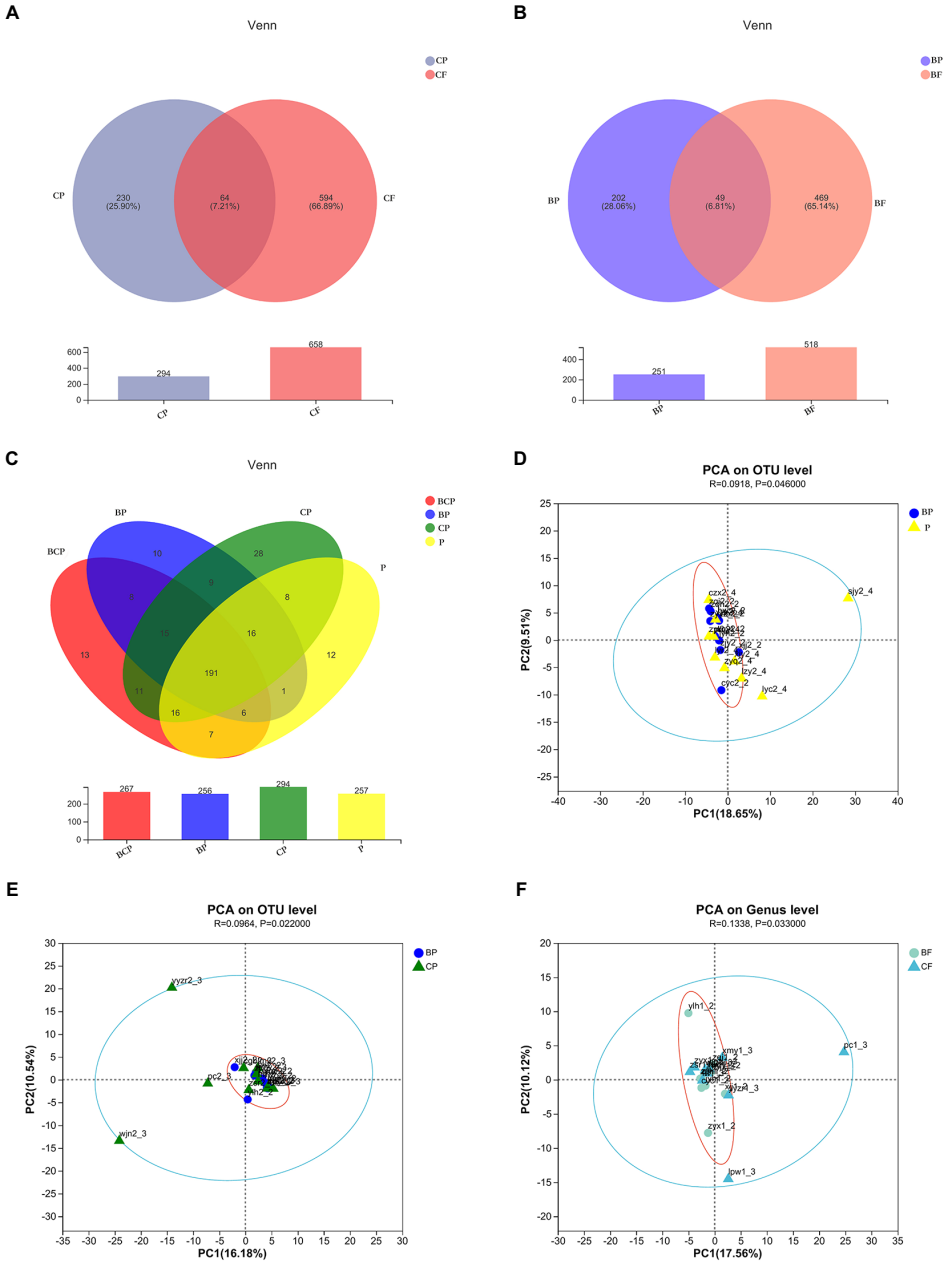
Venn diagrams depicting operational taxonomic unit (OTU) distributions in **(A)** CP and *CF* groups, **(B)** BP and BF groups, and **(C)** all plaque samples. Based on ANOSIM, PCA reveals differences in microbiome-community structures in **(D)** BP and P, **(E)** BP and CP, and **(F)** BF and *CF* groups.

A total of 4,340,246 high-quality reads were generated. Sequence OTU clustering and notation (at a 3% divergence level) identified 20 phyla, 36 classes, 80 orders, 141 families, 341 genera, 718 species, and 1,763 OTUs. The shared and unique OTUs in the subgroups are demonstrated in a Venn diagram (Figure 1). In both groups, most OTUs (*n* = 378) were preserved and shared between fecal groups, whereas 124 OTUs were unique to the BF group only (Figure 1D). In addition, 432 OTUs were detected in the *CF* group and F group, whereas 210 OTUs were unique to the *CF* group (Figure 1C). Also, 406 OTUs were common to the BCF group and F group, but 468 OTUs were detected only in the BCF group (Figure 1A). Notably, 191 OTUs were detected among all plaque subgroups (Figure 2C). In addition, 49 OTUs and 54 OTUs were present in plaque samples and fecal samples in EBS groups and caries groups, respectively (Figures 2A,B).

#### 3.3.2. Diversity of intestinal and plaque microbiota

**Figure 3:**
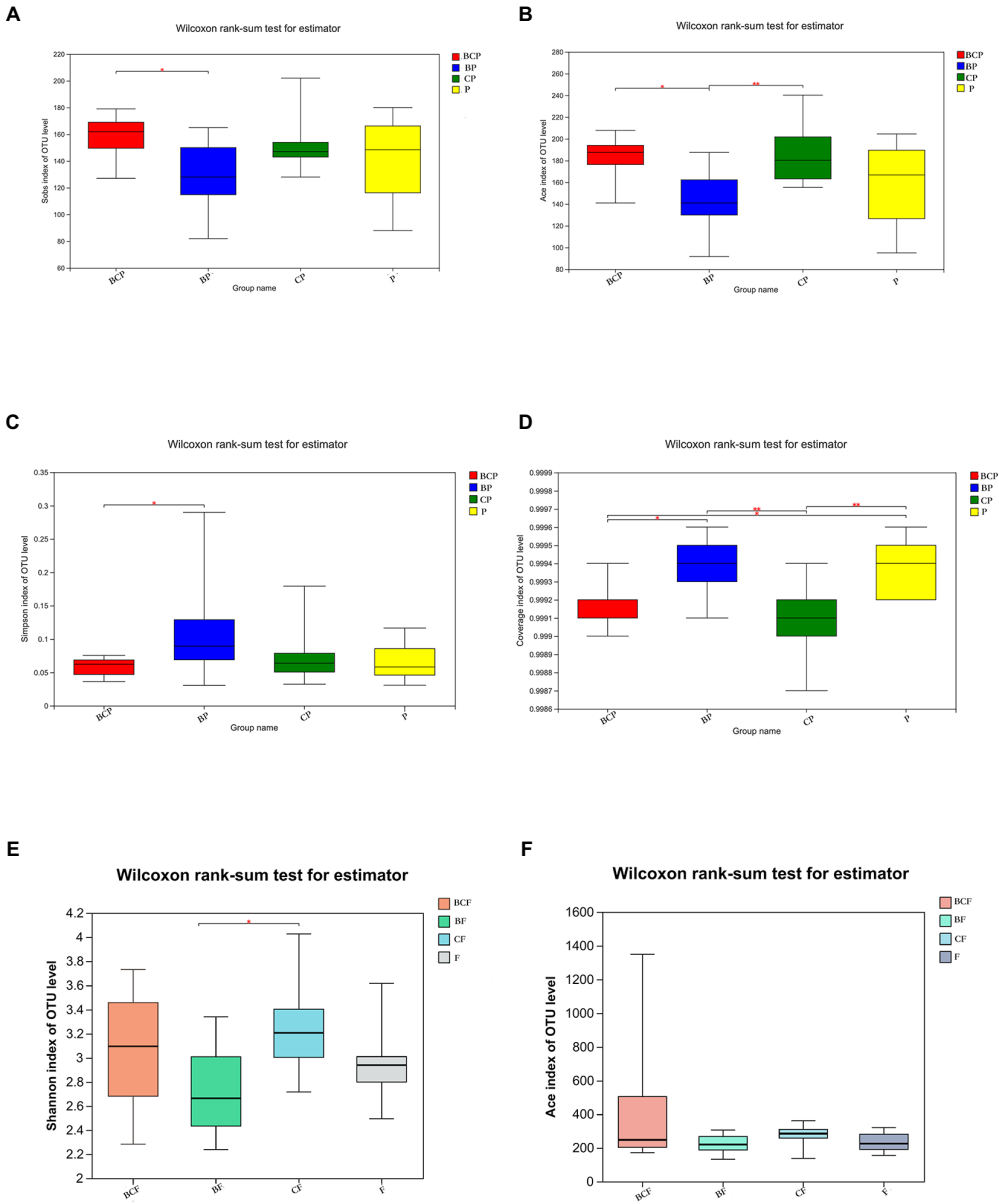
Comparison of α-diversity using the **(A)** Sobs Index, **(B)** Ace Index, **(C)** Simpson Index, **(D)** Coverage Index, **(E)** Shannon Index and **(F)** Ace Index. ^*^p < 0.05, ^**^p < 0.01, ^***^p < 0.001.

The observed species (Sobs) Index in the BCP group was higher than that in the BP group (Figure 3A), which indicated that samples from children who had both EBS and caries had more OTUs than samples from children with EBS but no caries. With regard to microbiota diversity, the Ace Index showed that samples from the BCP group and CP group had greater OTU richness than that of samples from the BP group (*p* < 0.05; Figure 3B). Comparison of Simpson Index data indicated that BP-group samples had significantly greater microbiome evenness than that of BCP-group samples (*p* < 0.05; Figure 3C). Significant differences were found in the Coverage Index in all pairwise comparisons (Figure 3D). The Shannon Index showed that *CF*-group samples had significantly greater microbiome diversity than BF-group samples (*p* < 0.05; Figure 3E). However, a significant difference was not observed between the other two groups in terms of community richness or diversity. Analyses of β-diversity revealed a difference in bacterial-community composition between groups. Principal component analysis (PCA) was used to analyze similarities and differences in the overall composition and structure of the microbiome between different groups. In BP and P groups, PC1 could explain 18.65% of the variation observed, whereas PC2 could explain 9.51% of the variation (Figure 2D). In BP and CP groups, PC1 could explain 16.18% of the variation and PC2 could explain 10.54% of the variation (Figure 2E). In BF and *CF* groups, PC1 could explain 17.56% of the variation, and PC2 could explain 10.12% of the variation (Figure 2F).

#### 3.3.3. Similarity and dissimilarity of bacterial compositions

**Figure 4:**
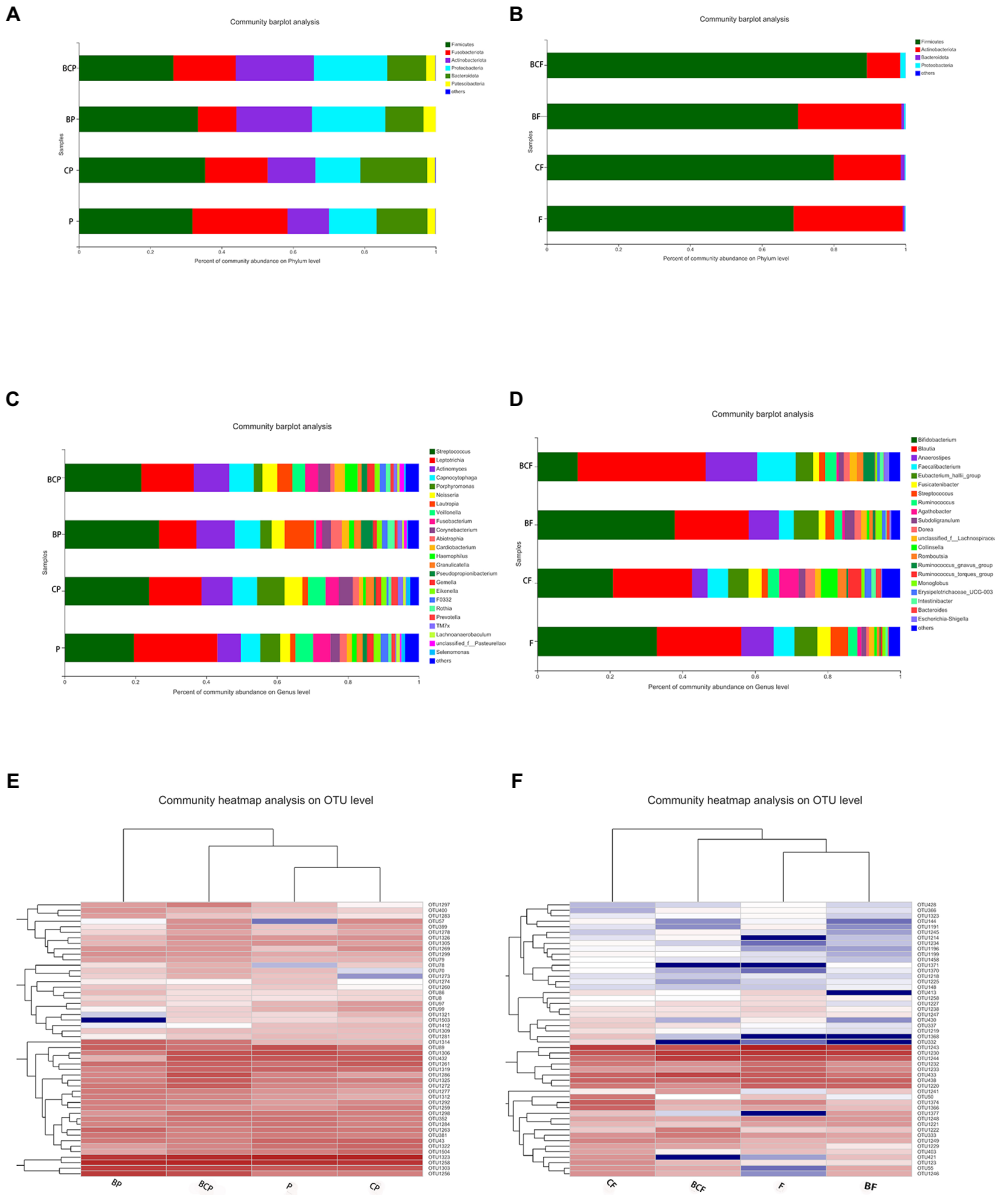
Distribution of predominant bacteria at the phylum level in **(A)** plaque samples and **(B)** fecal samples. Distribution of predominant bacteria at the genus level in **(C)** plaque samples and **(D)** fecal samples. **(E)** Distribution of the top-50 most abundant genera among plaque samples. Heatmaps were created using hierarchical clustering based on the (Bray–Curtis) distance. Columns represent the sample identifier, and rows represent the predominant bacterial genera. Variables of clustering are presented on the vertical axis. The relative values for bacterial genera are indicated by color intensity. **(F)** Bacterial distribution of the top-50 most abundant genera among fecal samples.

The top-50 abundant genera among plaque and fecal samples are displayed based on the heatmap of the genus-level plaque composition. The microbiota in dental plaque showed a more stable composition than the microbiota in the gut (Figures 4E,F). In terms of the OM, the six most abundant genera were *Streptococcus* (26.8%), *Actinomyces* (10.8%), *Leptotrichia* (10.6%), *Lautropia* (8.3%), *Capnocytophaga* (7.1%), and *Neisseria* (3.6%) in the BP group. Conversely, the six most abundant genera in the CP group were *Streptococcus* (23.8%), *Leptotrichia* (14.9%), *Actinomyces* (8.7%), *Porphyromonas* (7.7%), *Capnocytophaga* (7.0%), and *Veilonella* (5.1%). In the P group, *Leptotrichia* (23.7%), *Streptococcus* (19.6%), *Actinomyces* (6.5%), *Porphyromonas* (5.5%), *Capnocytophaga* (5.4%), and *Veilonella* (5.0%) were the most abundant genera (Figure 4C).

In fecal samples, *Blautia* (21.8%), *Bifidobacterium* (21.1%), *Eubacterium hallii* group (5.7%), *Faecalibacteriam* (5.6%), *Agathobacter* (5.5%), and *Collinsella* (4.4%) were the six most abundant genera in the *CF* group. In the BF group, the six most abundant genera were *Bifidobacterium* (38.0%), *Blautia* (20.5%), *Anaerostipes* (8.0%), *E. haillii* group (6.8%), *Facecalibacterium* (4.0%), and *Subdoligranulum* (2.7%; Figure 4D).

**Figure 5:**
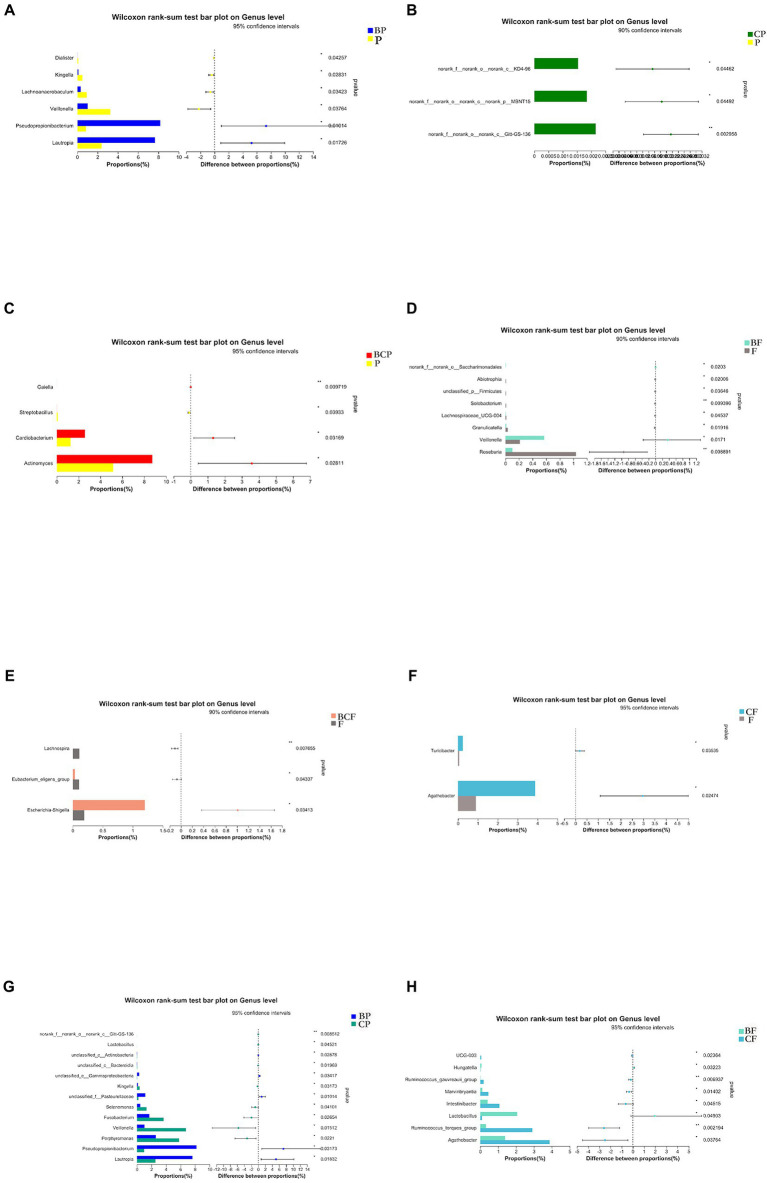
Plots based on the Wilcoxon rank-sum test at the genus level. Results of the comparison of **(A)** BP and P, **(B)** CP and P, **(C)** BCP and P, **(D)** BF and F, **(E)** BCF and F, **(F)** *CF* and F, **(G)** BP and CP, and **(H)** BF and *CF* groups. ^*^p < 0.05, ^**^p < 0.01, ^***^p < 0.001.

A specific method (linear discriminant analysis effect size) was employed to identify differentially enriched genera within groups. *Lautropia* and *Pesudopropionibacterium* species were highly abundant in the BP group, whereas *Veillonella*, *Lachnoanaerobaculum*, *Kingella*, and *Dialister* species were enriched significantly in the P group (Figure 5A). Comparison of the BP group and CP group revealed that *Lautropia* and *Pesudopropionibacterium* species were highly abundant in the BP group, whereas *Porphyromonas*, *Veillonella*, and *Fusobacterium* species were enriched in the CP group (Figure 5G). Comparison of the BCP group and P group revealed the relative proportions of *Actinomyces*, *Cardiobacterium*, and *Galella* species to be significantly greater in the former group (Figure 5C).

With respect to fecal samples, *Agathobacter* and *Turicibacter* species were enriched significantly in the *CF* group compared with the F group (Figure 5F), *Veillonella* species were significantly more common in the BF group than in the F group, and *Roseburia* species were more common in the F group than in the BF group (Figure 5D). *Agathobacter* and *Ruminococcus* species were more abundant in the *CF* group than in the BF group, whereas *Lactobacillus* species were more abundant in the BF group than in the *CF* group (Figure 5H). *Escherichia* and *Shigella* species were significantly more common in the BCF group than in the F group (Figure 5E).

#### 3.3.4. Environmental factors associated with bacterial communities in plaque and the intestine

**Figure 6:**
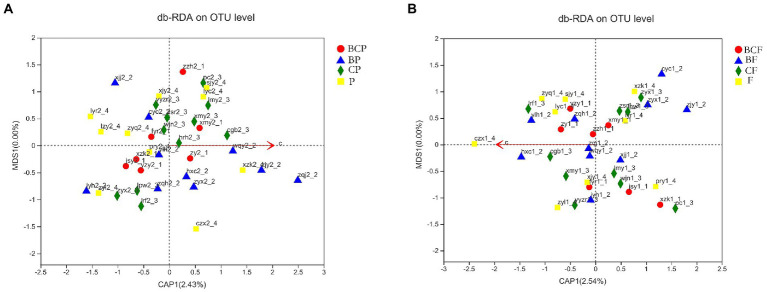
Relationship between the caries activity test (CAT) score bacterial-community structure. db-RDA plot at the species level in **(A)** plaque samples and **(B)** fecal samples.

db-RDA based on the Bray–Curtis distance matrix was undertaken to explore if other environmental factors (e.g., CAT score) had a role in the communities and composition of the GM and OM. OTU-level db-RDA results showed a relationship between the CAT score and bacterial-community structures in the plaque (r^2^ = 0.615, *p* < 0.001) and gut in enrolled children (Figure 6).

#### 3.3.5. Network analysis

**Figure 7:**
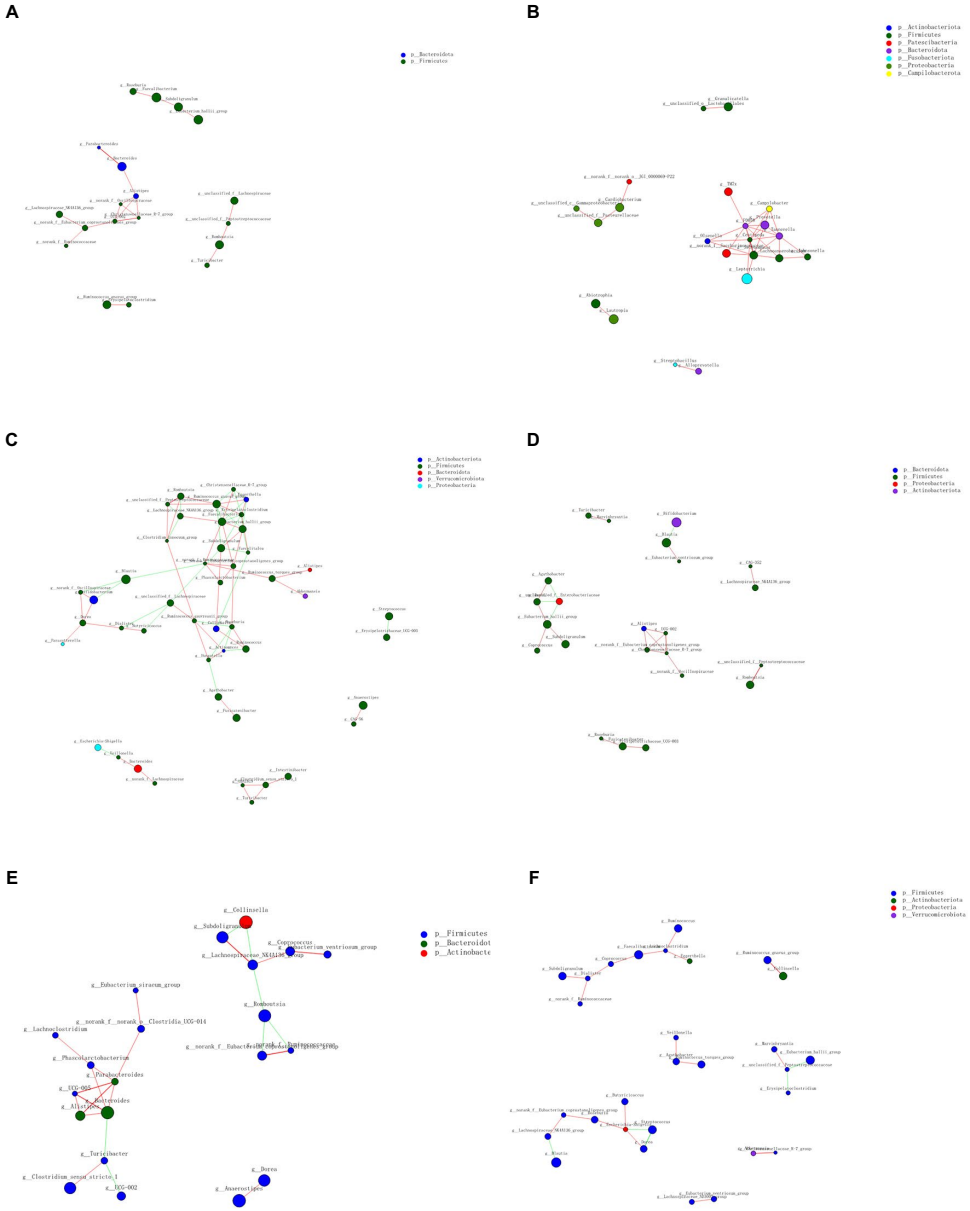
Network analysis showing interactions of the 50 richest genera (|Spearman coefficient| > 0.8, *p* < 0.01). Bacterial interactions in **(A)** all fecal samples, **(B)** all plaque samples, **(C)** BCF, **(D)** BF, **(E)** CF, and **(F)** F groups. The size of the node is proportional to the genera abundance. The node color corresponds to the phylum classification. The edge color represents positive (red) and negative (green) correlations.

**Figure 8:**
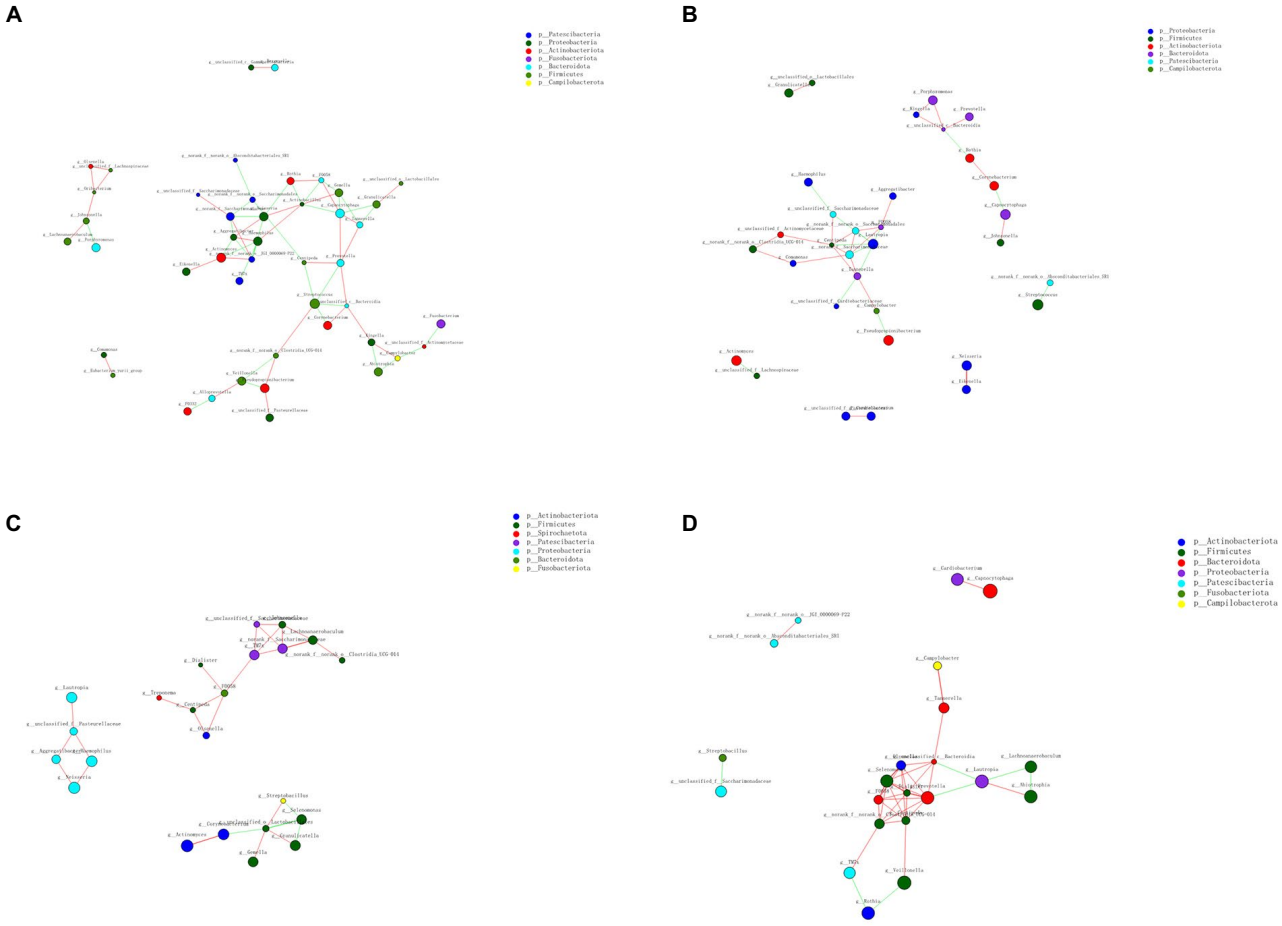
Network analysis revealed interactions of the 50 richest genera (|Spearman coefficientSpearmanCoef| > 0.8, *p* < 0.01). Bacterial interactions in **(A)** BCP, **(B)** BP, **(C)** CP, and **(D)** P groups.

Co-occurrence analysis was used to describe interactions among genera in different *niches*. A total of 158 genera were found to have complex interactions in dental plaque, whereas 275 genera were found to have complex interactions in fecal samples. The interactions of predominant genera are shown in Figures 7, 8. Among the intestinal microbiota, 21 genera displayed a high degree of association, and *Roserburia*, *Faecalibacterium*, *Subdoligranulum*, *Turicibacter*, *Parabacteroides, Bacteroides*, and *Alisipes* exhibited complicated interactions with each other. In the plaque microbiota, *TM7x, Campylobater, Leptotrichia*, and 19 other genera exhibited a positive correlation. Furthermore, different groups clearly showed different bacterial correlations. In the P group, the communities displayed more complex interactions (Figure 8). Meanwhile, compared with that in other groups, the microbiota in the BCF group exhibited more complex and aggregated relationships among each other (Figure 7).

## 4. Discussion

The EBS prevalence in the present study was 12.10%. In research conducted in other countries, the EBS prevalence was 2.4–18% (Zyla et al., 2015). EBS in permanent teeth was found in 16% of children living in the Philippines (Heinrich-Weltzien et al., 2009). Among those with primary dentition, the prevalence of EBS was 3.1% in Spanish children but 3.5% in 5-year-old Brazilian children (França-Pinto et al., 2012; Garcia Martin et al., 2013). In Tunisia, the EBS prevalence was 6.1% among children aged 3–5 years. Differences in diagnostic criteria, demographics, and sample size may account for the different values for EBS prevalence between countries. In a study conducted in Shanghai in 2013, the prevalence of EBS was 9.9% among 1,397 children with a mean age of 4.55 years. A study from Baotou Medical College (Baotou City, China) in 2019 revealed that 17.3% of 675 children aged 3–6 years had EBS. In 2015, of 718 children aged 5 years in Wenzhou, the EBS prevalence was 17.6%. These differences may be associated with socio-economic development, the awareness of parents regarding their children’s teeth, the age and eating habits of study participants, and sample size.

We showed an inverse correlation between the prevalence of EBS and caries. This result corresponds well with those of previous studies. In 2018, a meta-analysis demonstrated that patients with EBS are less likely to experience caries. Chen and colleagues revealed that children suffering from EBS had a lower prevalence of caries than that of healthy children (Chen et al., 2014). The association between black stains and caries in primary teeth has been researched in Tunisia and Brazil, with studies suggesting that EBS is a protective factor against caries in early childhood (França-Pinto et al., 2012; Elelmi et al., 2021).

We found a positive correlation between the CAT score and the mean dmft value (*p* = 0.023), which confirmed the conclusions reached in previous investigations. Xuan et al. (2017) concluded that the CAT (using Cariostat (Sankin)) could be employed to predict the caries risk of 3-year-old caries-free children. Thwin et al. (2022) used CAT scores to investigate the anti-caries effects of a school-administered fluoride varnish. CAT scores have also been employed to identify risk factors for caries in enamel and dentin in adolescents (Wang et al., 2020).

We discovered that EBS was more prevalent in children whose parents had a bachelor’s degrees or above and who had a greater awareness of the health of their own and their children’s teeth. Also, children who consumed Cow & Gate milk powder had a greater prevalence of EBS than those who drank other types of milk powder. Studies have focused mostly on the effects of eating dark-colored food, but few scholars have examined the importance of dairy products. In Brazil, EBS was more prevalent in children with lower family income levels at birth and whose mothers had low education levels (França-Pinto et al., 2012). Significant associations were not found between EBS and tooth-brushing, consumption of sweets, or bottle-feeding at night (Elelmi et al., 2021). In a study from Shanghai, factors for EBS included age, having been born in Shanghai, a higher level of parental education, less use of nursing bottles, consuming greater amounts of soy sauce, and a history of pneumonia (Chen et al., 2014). Variations in study regions or research participants hamper the ability to reach a uniform conclusion. Further assessments of various factors are needed, and larger sample sizes may be warranted.

Venn diagrams (Figure 1D) showed that most OTUs (n = 378) among fecal samples could be found between the BF group and F group, but 124 OTUs were unique to the BF group alone. A total of 432 OTUs were shared between the *CF* group and F group (Figure 1C), whereas 210 OTUs were unique to the *CF* group. Notably, when comparing the BCF group and F group, more unique OTUs were present in the former than in the latter. In addition, 468 (47.13%) OTUs were present only in the BCF group (Figure 1A). These results revealed a diverse bacterial community that differed between GM groups. Moreover, children who had both caries and EBS may have alterations in the GM. However, >70% of OTUs were consistent across plaque groups (Figure 2C), suggesting the existence of a more stable microbiota composition in dental plaque in all participants. The same microorganism structure was present in both plaque and fecal samples (Figures 2A,B). This result suggests a possible association between the OM and GM.

The BCP group had greater richness, but lower diversity, than the BP group. The CP group had greater richness than the BP group, and the *CF* group had higher diversity than the BF group. However, a high degree of similarity of the Shannon/Simpson and Chan/Ace indices were observed between the other groups. These data demonstrated that similar degrees of richness and diversity in the community of the OM and GM were present between the other two groups, respectively. These findings are in accordance with recent results, but the difference was not significant (Chen et al., 2019). However, Colak et al. (2013) observed that greater richness in the microbiome was present in samples of white-plaque biofilms samples, though there was no significant difference in the evenness index between EBS samples and white-plaque samples. Zhang et al. (2022) conducted 16S rRNA sequencing, and reported that the EBS group had high OTU richness, but lower evenness, than those in a group of healthy volunteers. Chen et al. (2021) showed that the EBS group exhibited greater bacterial richness than the control group. Therefore, a definitive conclusion is lacking, which could be attributed to the small sample size of our pilot study and variations in the number of teeth with EBS as well as the age, dietary habits, and lifestyle habits among study participants. Additional research involving larger study groups is required to reveal other environmental factors that affect dysbiosis in the microbiome community in children who have EBS and caries.

We observed only a few significant differences in α- and β-diversity. Nevertheless, we identified the potential organisms in the microbiota associated with EBS and caries in children. The most abundant genera in plaque were similar between groups, and our conclusions are consistent with those of previous studies. Specifically, the most abundant genera in plaque in patients with EBS were *Streptococcus*, *Actinomyces*, and *Leptotrichia* (Colak et al., 2013). The most abundant genera in the plaque of patients with caries were *Streptococcus*, *Leptotrichia*, *Actinomyces*, and *Porphyromonas*. In addition, the genera in the GM were similar, which indicated that the composition of the GM and OM communities was stable.

*Actinomyces* species belong to the resident OM of supragingival dental plaques. Slots and colleagues contended that EBS has a characteristic and relatively stable microflora dominated by various actinomycetes (Slots, 1974). Nagai et al. (2020) showed that *Actinomyces* can be considered the dominant genus associated with the formation of colored biofilms in children’s teeth. Zyla et al. (2015) concluded in a review that the microflora of EBS is dominated by *Actinomyces* species. Chen and colleagues also showed that the prevalence of *Cardiobacterium* species skewed from high to low, and that of *Porphyromonas* species skewed from low to high in the EBS group, *CF* (without EBS but with caries) group, and CS (healthy) group. Nevertheless, the exact mechanism of action has yet to be discovered. In our study, the BP group had the greatest proportion of *Actinomyces* species among all groups (10.82%), though the difference was not significant. However, children with both caries and EBS had more abundant concentrations of *Actinomyces* species and *Cardiobacterium* species than those in the control group. Therefore, determining the relationship between EBS and *Actinomyces* species and *Cardiobacterium* species would require more evidence based on larger studies with greater consideration of other environmental factors.

*Pseudopropionibacterium* is a new reclassification of the genus *Propionibacterium* proposed by Christian et al. (2016). In our study, *Pesudopropionibacterium* species were enriched significantly in the BP group, and *Porphyromonas* and *Fusobacterium* species were enriched in the CP group. These trends are similar to those of a previous study. Zhang et al. (2022) reported that the microbiome in their EBS group was characterized by various microbiome biomarkers, such as *Pseudomonas* species. Interestingly, Chen et al. (2021) found that the relative abundance of *Pseudopropionibacterium* species trended from high to low in the BS (EBS+, caries−), *CF* (EBS−, caries−), and CS (EBS−, caries+) groups, whereas that of *Porphyromonas* species trended from low to high in the BS, *CF*, and CS groups. *Fusobacterium* species and *Leptotrichia* species are associated with a high prevalence of caries (Liu et al., 2020; Celik et al., 2021).

The BP group having fewer *Porphyromonas* species and *Fusobacterium* species may have been the reason why EBS could be protective against caries. However, more studies are warranted to verify the role of *Pseudopropionibacterium* species in dysbiosis of the microbiome community for patients with EBS.

*Veillonella* species are biofilm-forming bacteria and related to disease (Corralo et al., 2021). Research has shown that *Veillonella* species are more abundant in individuals suffering from early-childhood caries compared with caries-free controls (Chen et al., 2019; Dame-Teixeira et al., 2021; Qudeimat, 2021). Sanz et al. (2017) revealed that *Veillonella* species contribute to the adhesion of *Streptococcus mutans* and consume the lactate generated by streptococci. With respect to the occurrence and development of periodontal diseases, *Veillonella* species provide adhesion sites for *Porphyromonas gingivalis* and boost the immune response (Luo et al., 2020). A separate review obtained identical results (Hua et al., 2020). In the present study, *Veillonella* species were significantly more abundant in CP and P groups than in the BP group, and promoted the adhesion of *S. mutans* and other cariogenic bacteria. This phenomenon can explain why the caries prevalence in the EBS-free group was significantly higher than that in the EBS group. In addition, *Veillonella* species are also present in the GM. In the present study, the BF group and F group could be distinguished by the presence of *Veillonella* species—that is, at the genus level, *Veillonella* was more common in the BF group than in the F group. Given that only a small proportion of *Veillonella* species were found in each group, they do not seem to be clinically important. However, to fully understand the relationship between the GM and human diseases, it is necessary to study even small proportions of the GM and their possible effects.

As novel Gram-negative, motile, and facultative anaerobic cocci, *Lautropia* species can be isolated from the dental plaque of gingivitis lesions (Lim et al., 2019). However, scholars have concluded that *Lautropia* species can be detected in supragingival dental plaques taken from healthy children (Shaddox et al., 2012; Hernandez et al., 2020). Papapanou et al. (2020) compared patients with periodontitis and found that the abundance of *Lautropia* species was significantly greater in healthy people. Qudeimat and colleagues discovered that *Lautropia* species were relatively more abundant in a caries-free group (Qudeimat et al., 2021). Chen et al. (2015) found that decreased carriage of *Lautropia* species was associated significantly with an increased risk of esophageal squamous cell carcinoma. Xu et al. (2021) showed that, in children given 100 g of probiotic yogurt daily for 1 year, concentrations of *Lautropia* species increased. *Lautropia* species were enriched in the dental plaques of children with EBS in the BP group, which may be the reason why BP-group membership correlated with a lower prevalence of caries. This finding may be associated with the protein in genes linked to bacterial motility, metabolism of linoleic acid, and flavonoid biosynthesis (Kim et al., 2018). Few studies have established a clear link between *Lautropia* species and pathogenicity.

Bacteria in the genus *Agathobacter* are anaerobic and Gram-positive. The main fermentation products of this genus are butyrate, acetate, hydrogen, and lactate (Hua et al., 2020). Butyrate is a crucial metabolite that provides energy for colonic epithelial cells to maintain intestinal-barrier and anti-inflammatory functions (Moon et al., 2020). Children with autism spectrum disorder and sleep disorders show a reduction in the abundance of *Agathobacter* species (Hua et al., 2020). Moon and colleagues showed that, in patients with systemic lupus erythematosus/Sjögren’s syndrome, the abundance of *Agathobacter* species was lower than that in the control group according to 16S rRNA sequencing (Moon et al., 2020). Yamamoto et al. (2019) showed that *Turicibacter* species were less abundant in patients on proton pump inhibitors than those in the control group, which tends to be a risk factor for hepatic encephalopathy or spontaneous bacterial peritonitis. Qudeimat et al. (2021) concluded that periodontitis correlates positively with the presence of *Ruminococcus* species, suggesting a key role of microbes in the exacerbating effect of the salivary microbiota on experimental colitis. *Ruminococcus* species have also been found to have important roles in gut inflammation (Schirmer et al., 2019). We discovered that bacteria of the genera *Agathobacter*, *Turicibacter*, and *Ruminococcus* were significantly more abundant in the GM in the caries group than in the EBS group. These results could have been because caries in children may have a relationship with intestinal dysbacteriosis and the intestinal barrier.

Among commensal bacteria, *Roseburia* species inhibit the activity of nuclear factor-kappa B (Inan et al., 2000) and can inhibit interleukin (IL)-17 secretion (Martin-Gallausiaux et al., 2016). *Roseburia* species can produce short-chain fatty acids, especially butyrate, which affects colonic motility, facilitates immunity maintenance, and has anti-inflammatory properties (Bousarghin et al., 2017). Butyrate can promote the oxidation and thermogenesis of fatty acids by inhibiting histone deacetylation in muscle, which increases energy expenditure (at least in part) by promoting mitochondrial functions in muscles (Zhanguo et al., 2009). *Roseburia* species are negatively associated with type-2 diabetes mellitus (Gurung et al., 2020). Their induction of IL-10 secretion may contribute to an improvement in glucose metabolism. *Roseburia intestinalis* can also increase the production of interleukin-22, an anti-inflammatory cytokine (Martin-Gallausiaux et al., 2016; Zhu et al., 2018). *Roseburia* species can restore insulin sensitivity and alleviate diabetes mellitus (Wang et al., 2014); moreover, they can promote the differentiation of regulatory T cells and induce transforming growth factor-β-based suppression of intestinal inflammation (Hoffmann et al., 2016; Shen et al., 2018; Zhu et al., 2018). In our pilot study, we showed that the abundance of *Roseburia* species in the control group was significantly higher than that in the EBS group. The role *Roseburia* species have in EBS requires further study.

Some *Lactobacillus* species inhibit the growth of cariogenic bacteria by producing antibacterial compounds or metabolites, competing with cariogenic bacteria for adhesion sites or co-aggregation, or by regulating the expression of genes related to cariogenic virulence (Wang and Zhou, 2012). Shi et al. (2020) concluded that *Lactobacillus* species can inhibit tooth decay by limiting the growth and virulence properties of *S. mutans*. *Lactobacillus* and *Bifidobacterium* species are most frequently used in, and have been efficacious therapeutic options for, the treatment/prevention of caries, periodontal diseases, urogenital infections, and gastrointestinal infections (Sales-Campos et al., 2019). Therefore, in recent years, probiotics containing *Lactobacillus* species have been assessed in the prevention and control of caries. We found that the abundance of *Lactobacillus* species was significantly greater in the BF group, which indicated that *Lactobacillus* species may be biomarkers in the GM for EBS to prevent caries.

*Escherichia* and *Shigella* species have been associated with a pro-inflammatory status (Morgan, 2013). Small et al. (2013) found that *Escherichia* species led to chronic and persistent infections with adherent and invasive characteristics. Those results are in accordance with data of studies revealing a positive correlation between changes in the abundance of *Escherichia* and *Shigella* species and changes in the levels of the pro-inflammatory molecules IL-6, (C-X-C motif) ligand 2, and NLR family pyrin domain containing-3 (Dame-Teixeira et al., 2021). Cattaneo and colleagues indicated that an increase in the abundance of *Escherichia* and *Shigella* species occurred in the progression from amyloid-β deposition to cognitive impairment in Alzheimer’s disease (Cattaneo et al., 2017). In another study, gut dysbiosis in Sjögren’s syndrome was observed. The authors reported a reduced abundance of bacteria of the genera *Bacteroides*, *Parabacteroides*, *Faecalibacterium*, and *Prevotella*, but an increased abundance of bacteria of the genera *Pseudobutyrivibrio*, *Escherichia*, *Shigella*, *Blautia*, and *Streptococcus* (Gong et al., 2014). de Paiva and colleagues determined that the abundance of *Escherichia* and *Shigella* species was increased significantly in patients with Sjögren’s syndrome (de Paivia et al., 2016). Moon and co-workers reported that oral or fecal bacteria from the genera *Haemophilus*, *Neisseria*, *Faecalibacterium*, *Romboutsia*, *Streptococcus*, *Gemella*, *Escherichia*, *Shigella*, and *Fusobacterium* may play an important part in tumor evolution (Moon et al., 2020). We found that the abundance of *Escherichia* and *Shigella* species was significantly greater in the GM in the EBS group than in the control group among children who had both caries and EBS. These results suggest that children who have both caries and EBS could exhibit a GM imbalance and peripheral inflammatory state. As such, the need for further research is evident.

Few studies have paid attention to the association between the GM community and oral disease. Sanz et al. (2017) showed that dysbiosis of the gastrointestinal microflora influenced the degree of demineralization of dental hard tissue, which may facilitate caries formation. Data have revealed that, in patients with more severe dysbiosis in the intestinal microflora, the prevalence and intensity of caries are significantly greater. However, data to explain how the GM influences EBS are lacking. More in-depth research is needed in the future.

Our study had four main limitations. First, the cross-sectional nature of our study can represent only the *status quo*, and we can merely speculate on the pathologic processes involved. Second, factors such as sex, lifestyle, aging, and saliva characteristics (pH, fluorine content) were not taken into account. Third, our samples were not classified by the degree of black stain, which might have influenced the results on microbiome community. Fourth, we did not analyze the elements in EBS plaque among participants.

However, our study provides, for the first time, evidence suggesting the involvement of the metabolic pathways of oral and intestinal microflora in EBS formation in primary dentition. As such, the detailed mechanisms were not clear and not explored in our study. We limited our study to bacteria in dental plaque and the GM, so future studies could focus on fungi and other oral microorganisms. The microbiome community in saliva and the specific mechanisms behind how the OM and GM can produce and influence EBS require more in-depth research to help find novel methods for the prevention and treatment of EBS.

## 5. Conclusion

The microbiome in dental plaque showed a more stable composition than that in the GM. Homologs were observed between the OM and GM. Among plaque groups, the control group had the most complex microbial environment. For dental plaque, children with both EBS and caries exhibited greater OTU richness but lower evenness compared with children with EBS without caries. The caries group showed greater richness in plaque samples and fecal samples. The BCF group had the largest number of OTUs and exhibited the most complex relationships among all fecal groups. At the genus level, a greater relative abundance of *Actinomyces* and *Cardiobacterium* was observed in the BCP group, whereas *Lautropia* and *Pesudopropionibacteriumin* were observed more in the BP group (*p* < 0.05). GM groups were characterized by various microbiome biomarkers, such as *Escherichia* and *Shigella* species in the BCF group, *Veillonella* species in the BF group, and *Agathobacter* and *Ruminococcus* species in the *CF* group. This information can be used as a basis for understanding the bacteria involved in EBS formation.

Our results provide new ideas for the prevention and treatment of EBS and caries. In the future, the study sample size should be enlarged. The pH of saliva and other characteristics should be considered. EBS should be graded to explore its components and the dominant bacteria present. After the dominant bacteria have been cultured *in vitro*, animal experiments should be conducted to confirm the oral- and intestinal-dominant bacteria of EBS so as to obtain the corresponding prevention-and-treatment measures.

## Data availability statement

The 16S rRNA gene sequencing data presented in the study are deposited in the Sequence Read Archive (SRA) database, accession number PRJNA901537.

## Ethics statement

The studies involving human participants were reviewed and approved by institutional ethics committee of Stomatological Hospital, which is affiliated with Wenzhou Medical University. Written informed consent to participate in this study was provided by the participants’ legal guardian/next of kin. The animal study was reviewed and approved by institutional ethics committee of Stomatological Hospital, which is affiliated with Wenzhou Medical University.

## Author contributions

LZ and TC conceived the study and designed the experiments. LZ, TC, and PX collected samples. LZ carried out the experiments and analyzed the data. LZ wrote the manuscript. LZ, TC, PX, and YM participated in the epidemiological investigation. LW and JW edited and approved the final version of the manuscript. All authors contributed to writing the manuscript and approved the submitted version.

## Funding

This work was supported by the Science and Technology Project Program of Wenzhou (Y20210116 and Y20210120) and the Education Program of Zhejiang Province (Y202147090).

## Conflict of interest

The authors declare that the research was conducted in the absence of any commercial or financial relationships that could be construed as a potential conflict of interest.

## Publisher’s note

All claims expressed in this article are solely those of the authors and do not necessarily represent those of their affiliated organizations, or those of the publisher, the editors and the reviewers. Any product that may be evaluated in this article, or claim that may be made by its manufacturer, is not guaranteed or endorsed by the publisher.
