# Supplementary material for: Characterization of the oral microbiome and gut microbiome of dental caries and extrinsic black stain in preschool children
Source: Front Microbiol. 2023 Mar 31;14:1081629. doi: 10.3389/fmicb.2023.1081629 (PMC10103904; doi:10.3389/fmicb.2023.1081629)
Supplement: Supplementary file 1 [file Data_Sheet_1.PDF]

Supplementary Material

Characterization of the Oral Microbiome and Gut Microbiome of Dental Caries and Extrinsic Black Stain in Preschool Children

Luoyuan Zheng, Tingting Cao, Puling Xiong, Yulian Ma, Limin Wei*, Jianfeng Wang*

*** Correspondence:** Limin Wei: lwei525@wmu.edu.cn; Jiangfeng Wang: jwang525@wmu.edu.cn

1 Supplementary Data

STROBE checklist

	Item No	Recommendation	
**Title and abstract**	1	(*a*) Indicate the study's design with a commonly used term in the title or the abstract	√
		(*b*) Provide in the abstract an informative and balanced summary of what was done and what was found	√
**Introduction**			
Background/rationale	2	Explain the scientific background and rationale for the investigation being reported	√
Objectives	3	State specific objectives, including any prespecified hypotheses	√
**Methods**			
Study design	4	Present key elements of study design early in the paper	√
Setting	5	Describe the setting, locations, and relevant dates, including periods of recruitment, exposure, follow-up, and data collection	√
Participants	6	(*a*) Give the eligibility criteria, and the sources and methods of selection of participants	√
Variables	7	Clearly define all outcomes, exposures, predictors, potential confounders, and effect modifiers. Give diagnostic criteria, if applicable	not discussed
Data sources/ measurement	8*	For each variable of interest, give sources of data and details of methods of assessment (measurement). Describe comparability of assessment methods if there is more than one group	√
Bias	9	Describe any efforts to address potential sources of bias	√
Study size	10	Explain how the study size was arrived at	√
Quantitative variables	11	Explain how quantitative variables were handled in the analyses. If applicable, describe which groupings were chosen and why	√

Statistical methods	12	(*a*) Describe all statistical methods, including those used to control for confounding	√
		(*b*) Describe any methods used to examine subgroups and interactions	√
		(*c*) Explain how missing data were addressed	not discussed
		(*d*) If applicable, describe analytical methods taking account of sampling strategy	√
		(*e*) Describe any sensitivity analyses	not discussed
**Results**			
Participants	13*	(a) Report numbers of individuals at each stage of study—eg numbers potentially eligible, examined for eligibility, confirmed eligible, included in the study, completing follow-up, and analysed	√
		(b) Give reasons for non-participation at each stage	not discussed
		(c) Consider use of a flow diagram	not discussed
Descriptive data	14*	(a) Give characteristics of study participants (eg demographic, clinical, social) and information on exposures and potential confounders	not discussed
		(b) Indicate number of participants with missing data for each variable of interest	not discussed
Outcome data	15*	Report numbers of outcome events or summary measures	√
Main results	16	(*a*) Give unadjusted estimates and, if applicable, confounder-adjusted estimates and their precision (eg, 95% confidence interval). Make clear which confounders were adjusted for and why they were included	not discussed
		(*b*) Report category boundaries when continuous variables were categorized	not discussed
		(*c*) If relevant, consider translating estimates of relative risk into absolute risk for a meaningful time period	not discussed
Other analyses	17	Report other analyses done—eg analyses of subgroups and interactions, and sensitivity analyses	√
**Discussion**			
Key results	18	Summarise key results with reference to study objectives	√
Limitations	19	Discuss limitations of the study, taking into account sources of potential bias or imprecision. Discuss both direction and magnitude of any potential	√

		bias	
Interpretation	20	Give a cautious overall interpretation of results considering objectives, limitations, multiplicity of analyses, results from similar studies, and other relevant evidence	√
Generalisability	21	Discuss the generalisability (external validity) of the study results	√
**Other information**			
Funding	22	Give the source of funding and the role of the funders for the present study and, if applicable, for the original study on which the present article is based	√

Questionnaire survey on dental caries and EBS in children

In children, dental caries not only affects their oral health but can also lead to poor growth, which can affect their mental health. According to the survey by the Stomatological Hospital affiliated with Wenzhou Medical University, caries risk among children aged 3–6 years in Wenzhou is higher than the national average. Recently, our hospital conducted a survey on whether children's dental caries and other oral diseases are related to diet, parents' awareness of oral health, and other factors. We hope you can fill out the following questionnaire carefully. Thank you for your cooperation.

1. The campus where your child is enrolled _________

2. Name and class of your child _________

3. Your relationship with your child _________

   Your age _________

   How many children do you have? _________

   Her or his birth order

   - First child
   - Second child

4. Who is responsible for the child's daily life?

   - Parents
   - Grandparents
   - Someone else

5. Your level of education

   - Below high-school-education graduate
   - High-school-education graduate
   - Technical-school graduate or above

- Bachelor's degree or above

6. Who is the main monitor of your child's tooth-brushing?

- Himself/herself
- Parents
- Both

7. How often does your child brush his/her teeth?

- <1/day
- 1/day
- ≥2/day

8. The correct way for preschoolers to brush their teeth is:

- Arc brushing method
- Pap brushing method

9. Children should not use fluoride toothpaste.

- Yes
- No

10. How often do you brush your teeth?

- 1/day
- ≥2/day

11. How long do you need to brush your teeth?

- <1 min
- 1–2 min
- ≥2 min

12. How often do you change your toothbrush?

- <3 months
- 3–6 months
- ≥6 months

13. How often do you use dental floss?

- Everyday
- Often
- Sometimes
- Never

14. Do you have regular oral checkups?

- Yes
- No

15. Do you know about dental plaque?

- Yes
- No

16. Do you know about Fluor Protector?

- Yes
- No

17. Have you ever taken your child to the hospital to apply fluoride?

- Yes
- No

18. Do you know about pit and fissure sealing?

- Yes
- No

19. Does your child have dental caries and has he/she been treated?

- Have caries but have not been treated
- Have caries and have been treated
- No caries

20. Which of the following dangers of dental caries do you know about?

- Affects chewing
- Affects pronunciation
- Affects facial appearance
- Affects growth and development of the face
- Affects general health
- Affects mental health
- Causes pain

21. How long does your child spend eating a meal?

- <20 min
- 20–40 min
- 40–60 min
- >60 min

22. Does your child have a mouth-breathing habit?

- Often
- During sleeping
- When he/she has a cold
- I don’t know

23. Does your child have rhinitis?

- Yes, and has been treated
- Yes, but has not been treated
- No
- I don’t know

24. Does your child have bad oral habits?

- Bites upper lips
- Bites lower lips
- Tongue habit
- Sucks finger
- Sucks pens
- None

25. Does your child snore?

- Never
- Sometimes
- Often
- Always

26. Has your child been to the orthodontic department of a hospital?

- Yes, but has not been treated
- Yes, and has been treated
- Never

27. Do you or your family have a reverse overjet or overbite?

- Parents have reverse overjet, but children don’t
- Both parents and children have reverse overjet
- Parents have an overbite, but children don’t
- Both parents and children have an overbite
- No

28. Do you or your family have mandibular retrusion?

- Parents have mandibular retrusion, but children don’t

- Both parents and children have mandibular retrusion
- Parents don't have mandibular retrusion, but children have
- No

29. Does your child have anemia?

- Yes
- No
- I don't know

30. What are the brand(s) of milk powder your child drank (or is drinking) at different stages of life?

- First stage _________
- Second stage _________
- Third stage _________
- Other _________

31. During which of the following stages of life did (or does) your child drink milk powder?

- 0–6 months
- 6–12 months
- 12–36 months
- >36 months

32. How often does your child drink milk powder?

- Never
- 1–2/day
- 3–4/day

33. How much milk powder does your child need per month?

- Never
- ≤1 container
- 1–2 container(s)
- 2–3 containers
- ≥3 containers

34. How many milliliters of milk powder does your child drink each time?

- ≤100 mL
- 101–200 mL
- 201–300 mL
- ≥300 mL
- Other

35. Does your child eat cheese?

- Eats cheese often
- Eats cheese sometimes
- Other

2 Supplementary Figures and Tables

2.1 Supplementary Tables

SUPPLEMENTARY TABLE 1 Association between EBS and parent variables

Question	Variable		EBS prevalence	*p*
Parents' education level	≤15 years		5.57%	0.034[*]
	>15 years		9.25%	
Parents' prevention awareness	Knows of Fluor Protector		8.19%	0.0317[#]
	Does not know of Fluor Protector		6.22%	
Parents' hygiene habits	How often do you change toothbrush?	<3 months		0.814
		3–6 months		
	How often do you use dental floss?	Everyday	11.02%	0.874
		Often	10.32%	
		Sometimes	8.76%	
		0	10.18%	

	Regular oral checkups?	Yes	14%	<0.01**
		No	6.26%	
	Knows about dental plaque?	Yes	9.60%	0.738
		No	10.38%	
	Knows about pit and fissure sealing?	Yes	10.23%	
		No	8.21%	0.4
Number of children	1		10.80%	0.719
	≥2		9.90%	
Birth order	First child		8.89%	0.697
	Second child		10.28%	

**Compared with children whose parents have >15 years of education, $p < 0.05$.*

#Compared with children whose parents do not have knowledge of Fluor Protector, $p < 0.05$.

***Compared with children whose parents do not have regular oral checkups, $p < 0.01$.*

SUPPLEMENTARY TABLE 2 Association between EBS and pediatric variables

**Question**	**Variable**	**EBS prevalence**	***p***
Brushing	Themselves	13.99%	0.349
	Parents	8.97%	
	Both	11.28%	

Brushing frequency	1/day		8.11%	
	≥2/day		10.06%	
Anemia	Yes		6.98%	0.836
	No		8.37%	
Eating habits	Eats cheese often		7.89%	1
	Eats cheese sometimes		7.92%	
	Drinks Cow & Gate™ milk powder		14.67%	0.029*
	Drinks other milk powder		7.46%	
	Drinks milk powder		7.81%	1
	Drinks breast milk		7.14%	
	Meal time	<20 min	7.76%	0.278
		20–40 min	11.15%	
		40–60 min	10%	
Frequency of physician visits	Uses Fluor Protector often		9.48%	0.17
	Uses Fluor Protector sometimes		6.94%	
	Receives oral treatment often		4.27%	<0.01*
	Receives oral treatment sometimes		8.46%	
Daily habits	Mouth breathing	Often	3.45%	0.466

	During sleeping	10.61%	
	When having cold	9.93%	
Bad oral habits	Bites upper lips	5.13%	0.264
	Bites lower lips	4.29%	
	Tongue habit	14.75%	
	Sucks finger	14.75%	
	Sucks pens	11.76%	
	Never	11.36%	
Snoring	Never	9.02%	0.727
	Sometimes	10.66%	
	Often	9.30%	

**Compared with children who do not drink Cow & Gate™ milk powder, $p < 0.05$.*

***Compared with children who receive oral treatment sometimes, $p < 0.01$.*

2.2 Supplementary Figures

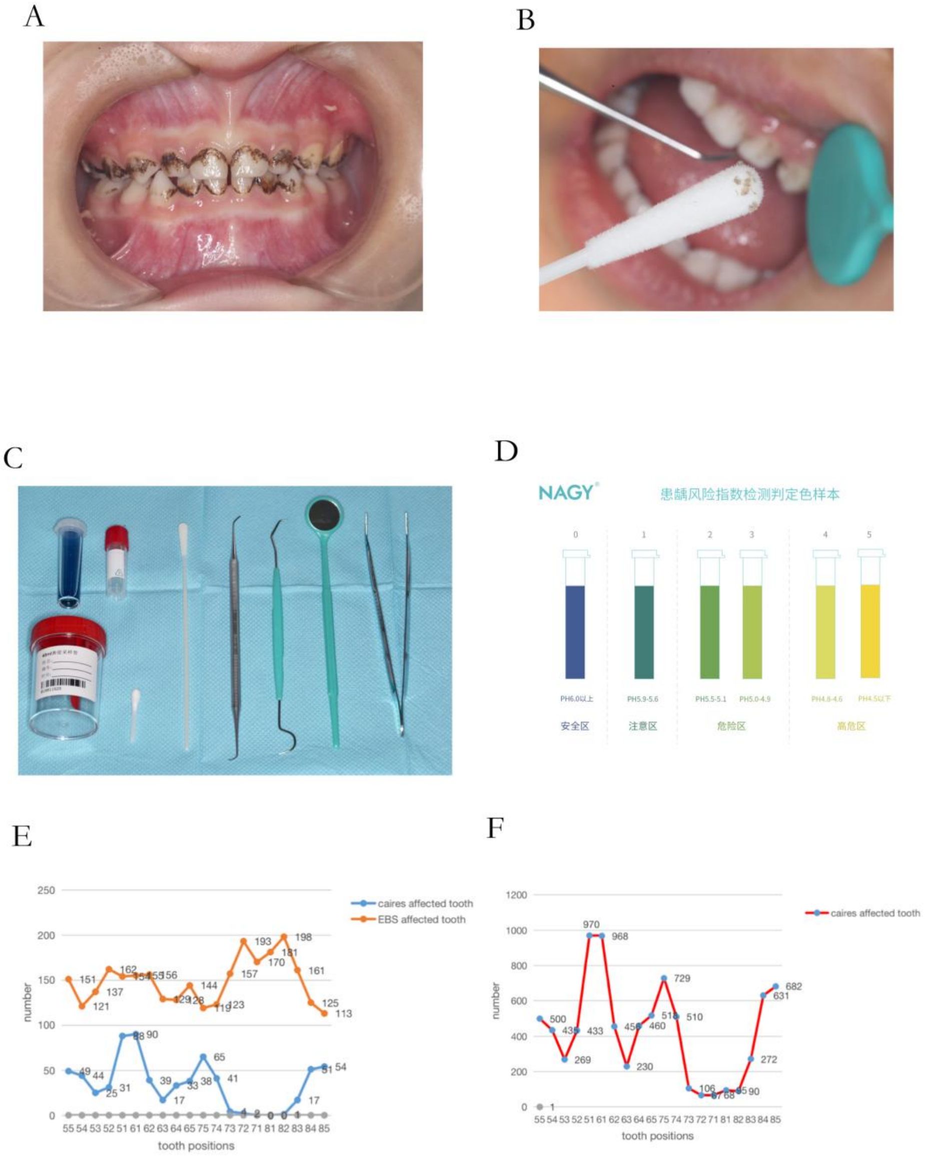

SUPPLEMENTARY FIGURE 1 (A) Primary dentition with EBS. Note the dark-pigmented extrinsic substance on the buccal and/or lingual surfaces of the teeth near the gingival margin. (B, C) Experimental supplies and process. (D) CAT scores are presented in six levels according to different colors. (E) The teeth and surfaces most affected by EBS were the mandibular anterior teeth and their lingual surfaces. (F) The teeth most affected by caries were the maxillary anterior teeth.
